# Texas Congress of Mothers

**Published:** 1914-12

**Authors:** 


### Texas Congress of Mothers.

The Texas Congress of Mothers, which met in San Antonio last month, closed its most successful year's work. Next to the home and the school this Congress is the greatest factor in the State for the betterment of the child. It is composed of mothers seeking instruction to prepare themselves for more efficient motherhood. To the extent that their work subserves the rearing of better children, it is a eugenistic movement. Eugenics includes environment as a necessary soil to develop the better germ plasm it would evolve through the proper understanding of mating; then what better opportunity could the eugenist wish for than the organized, enlightened motherhood of the country? If the Congress can see clearly that this is its *real* work, and will not be drawn off into other endeavors of woman's work, it will demand and receive a greater measure of support and encouragement from the public.

This is an age of specialism, and the greatest specialty of all is the rearing of better babies. This alone should fill the heart and hands of any woman or organization of women. Keep to that high aim and your efforts will be blessed.

The Congress is specially to be congratulated on its good fortune to have arranged with the University Extension Bureau and the College of Industrial Arts to furnish the various clubs throughout the State systematic courses of adapted studies in the various interests that affect the child and motherhood. This is a real advancement in the right direction, and displaces resoluting with actual doing. Who can foretell the dynamic power such a work can create in a few years of earnest persistency? Motherhood is the crowning glory of womanhood. Then hats off to the organized motherhood of Texas!

